# Crystal structures of bis­[(9*S*,13*S*,14*S*)-3-meth­oxy-17-methyl­morphinanium] tetra­chlorido­cobaltate and tetra­chlorido­cuprate

**DOI:** 10.1107/S2056989016019939

**Published:** 2017-01-01

**Authors:** Eric Gauchat, Alexander Y. Nazarenko

**Affiliations:** a Chemistry Department, SUNY Buffalo State, 1300 Elmwood Ave, Buffalo, NY 14222, USA

**Keywords:** crystal structure, dextromethorphan, tetra­chlorido­cobaltate, tetra­chlorido­cuprate, N—H⋯Cl hydrogen bonds

## Abstract

(9*S*,13*S*,14*S*)-3-Meth­oxy-17-methyl­morphinan (dextromethorphan) forms two isostructural salts with (*a*) tetra­chlorido­cobaltate, namely bis­[(9*S*,13*S*,14*S*)-3-meth­oxy-17-methyl­morphinanium] tetra­chlorido­cobaltate, (C_18_H_26_NO)_2_[CoCl_4_], and (*b*) tetra­chlorido­cuprate, namely bis­[(9*S*,13*S*,14*S*)-3-meth­oxy-17-methyl­morphinanium] tetra­chlorido­cuprate, (C_18_H_26_NO)_2_[CuCl_4_]. The distorted tetra­hedral anions are located on twofold rotational axes. The dextromethorphan cation can be described as being composed of two ring systems, a tetra­hydro­naphthalene system *A*+*B* and a deca­hydro­isoquinolinium subunit *C*+*D*, that are nearly perpendicular to one another: the angle between mean planes of the *A*+*B* and *C*+*D* moieties is 78.8 (1)° for (*a*) and 79.0 (1)° for (*b*). Two symmetry-related cations of protonated dextromethorphan are connected to the tetra­chlorido­cobaltate (or tetra­chlorido­cuprate) anions *via* strong N—H⋯Cl hydrogen bonds, forming neutral ion associates. These associates are packed in the (001) plane with no strong attractive bonding between them. Both compounds are attractive crystalline forms for unambiguous identification of the dextromethorphan and, presumably, of its optical isomer, levomethorphan.

## Abstract 2

In the crystal structures of title compounds, two identical protonated dextromethorphan cations are connected to tetra­chlorido­cobaltate (or tetra­chlorido­cuprate) anions *via* strong N—H⋯Cl hydrogen bonds, forming neutral ion associates

## Chemical context   

Seemingly innocuous and common over-the-counter drugs have a wide range of uses to treat illness and relieve pain, but they can also lead to long-term abuse and fatalities. Dextromethorphan (systematic name (9*S*,13*S*,14*S*)-3-meth­oxy-17-methyl­morphinan**)** is a member of the *Morphinan* class of naturally occurring and semi-synthetic psychoactive drugs, chemically similar to morphine, codeine and oxycodone, and differing from these only by a few functional groups. It is commonly found in many cold and cough medicines. In high concentrations, dextromethorphan has effects similar to phencyclidine and ketamine, a dissociative anesthetic, which is known to induce visual hallucinations and a heightened sense of perceptual awareness (Bruera & Portenoy, 2010 ▸). The similarity to well-known substances of abuse that are highly controlled makes dextromethorphan an attractive target for recreational ingestion and purification from over-the-counter products.

Cobalt(II) compounds have been employed in color tests for alkaloid detection: *e.g*., the Scott reagent (Cole, 2003 ▸). However, color reactions are usually not very specific and may lead to numerous false positives. To complicate the issue, levomethorphan, an optical isomer of dextromethorphan, is a strong opiate drug and is restricted like morphine in the US and many other countries. Therefore, usual NMR and MS identification may be insufficient for clear identification of dextromethorphan and levomethorphan.

We suggest that easy-to-grow crystals of alkaloid metal complexes may provide a suitable analytical approach for unambiguous identification. As a part of this study, we report the crystal structures of two such compounds here.
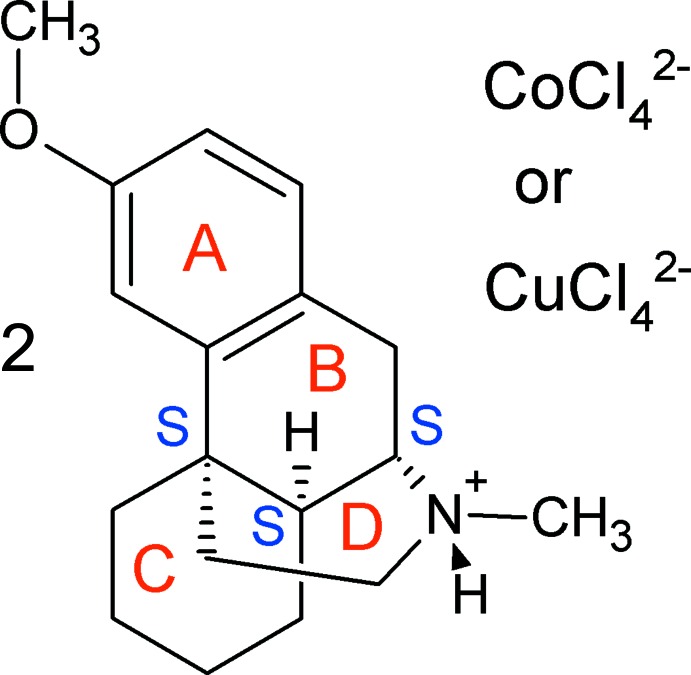


## Structural commentary   

**Figure 1:**
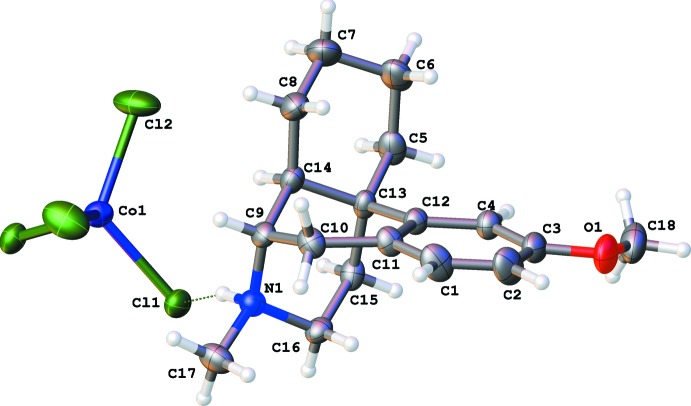
The numbering scheme of the dextromethorphan tetra­chlorido­cobaltate complex (*a*) with displacement ellipsoids drawn at the 50% probability level.

**Figure 4:**
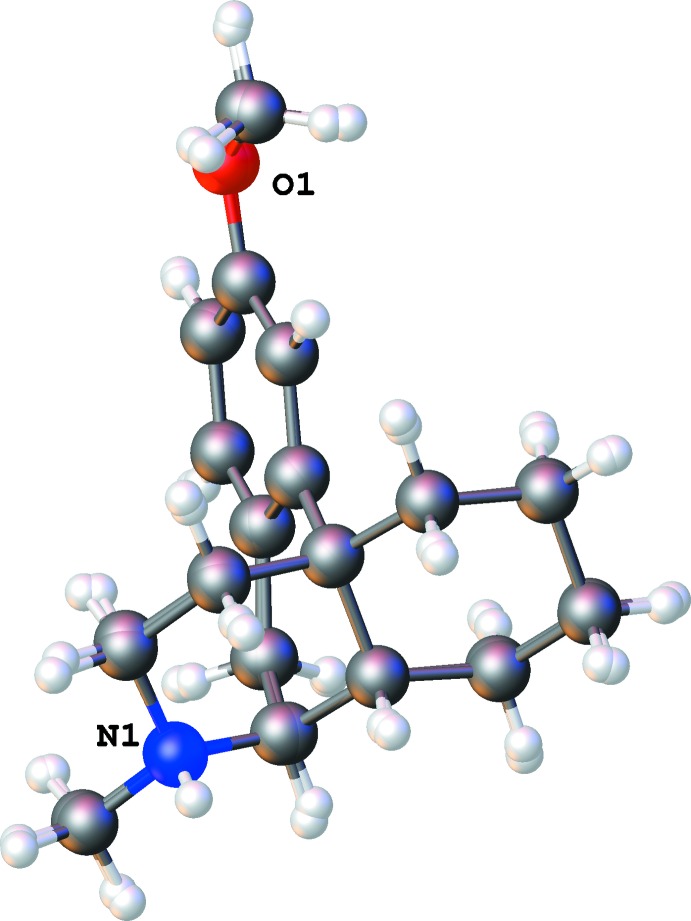
Overlay of the protonated dextromethorphan cation (*a*) and the dextromethorphan mol­ecule (refcode XAPTAK01).

The protonated dextromethorphan cations are nearly identical (Figs. 1 ▸–3 ▸ ▸). In both cases, protonation as well as inter­action with the tetra­chlorido­cobaltate or tetra­chlorido­cuprate anions does not affect the geometry of the methorphan ring system (Fig. 4 ▸), leaving the shape of the organic mol­ecule intact. The derived mol­ecular dimensions within both structures are unexceptional and consistent with those known for similar mol­ecules (Gylbert & Carlström, 1977 ▸).

There are four six-membered rings in a dextromethorphan mol­ecule. The aromatic ring *A* is practically planar with deviations less than 0.01 Å in all cases. The cyclo­hexene ring *B* can be described as a half-chair shifted towards an envelope conformation: atoms C10, C11, C12 and C13 are adjacent to the aromatic ring and therefore almost planar while C9 and C14 deviate from this plane in opposite directions: C9 − 0.191 (6) Å (*a*) and −0.173 (8) Å (*b*); C14 + 0.553 (5) Å (*a*) and +0.562 (8) Å (*b*). This half-chair conformation is known (Ibberson *et al.*, 2008 ▸) for the unsubstituted cyclo­hexene mol­ecule in the solid state.

The cyclo­hexane *C* and piperidine *B* rings both have chair conformations. These two rings are nearly coplanar, with the angles between their mean planes being 7.8 (1)° (*a*) and 8.2 (2)° (*b*). As a result, the dextromethorphan cation can be described as two ring systems *A*+*B* and *C*+*D*, being nearly perpendicular to each other: the angle between the mean planes of the *A*+*B* and *C*+*D* moieties is 78.8 (1)° for (*a*) and 79.0 (1)° for (*b*).

The tetra­chlorido­cobaltate and tetra­chlorido­cuprate anions both have a distorted tetra­hedral geometry. In the cobalt complex, the Cl1—Co1—Cl2 angle is flattened to 116.59 (3)°, while in the copper analogue the Cl2—Cu1—Cl1 angle is 129.04 (4)°. The larger deviation from tetra­hedral geometry in the copper(II) compound is possibly due to the Jahn–Teller effect, as packing effects should be similar in both compounds.

## Supra­molecular features   

**Figure 5:**
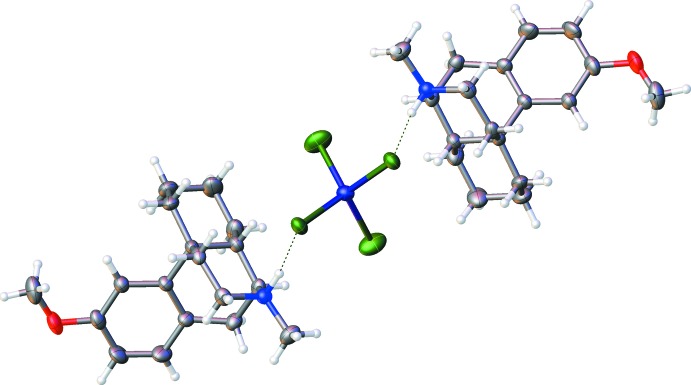
Two dextromethorphan cations forming an ion associate with the tetra­chlorido­cobaltate dianion. Hydrogen bonds are drawn as dashed lines.

**Figure 6:**
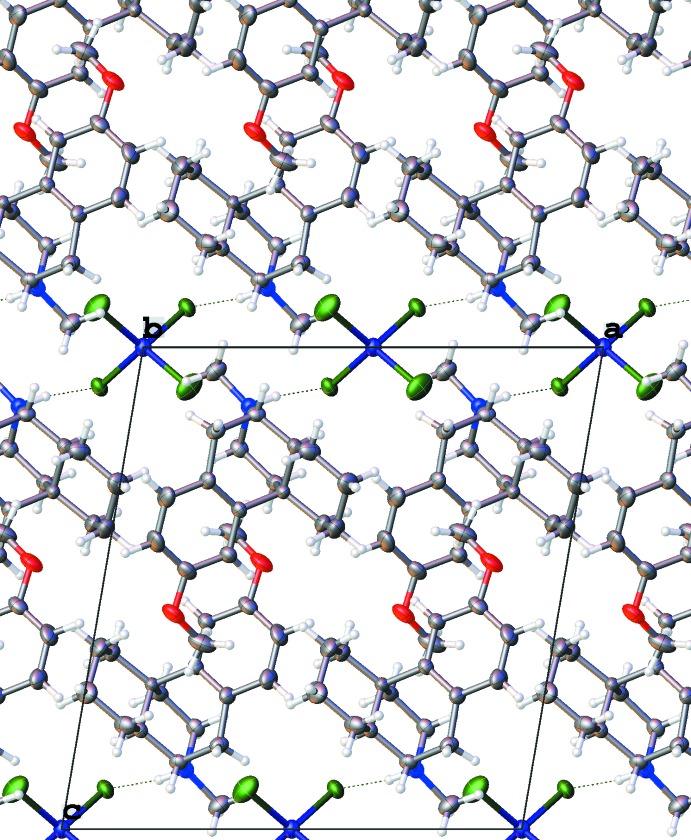
Packing diagram of the ion associates in structure (*a*), viewed along [010].

The tetra­chlorido­cobaltate and tetra­chlorido­cuprate anions are located on twofold rotational axes. Two identical protonated dextromethorphan cations are connected to tetra­chlorido­cobaltate (or tetra­chlorido­cuprate) anions *via* strong N—H⋯Cl hydrogen bonds (Tables 1 ▸ and 2 ▸), thus forming neutral ion associates (Fig. 5 ▸). These associates are packed into layers in the (001) plane (Fig. 6 ▸) with no strong attractive bonding between them. Methyl groups adjacent to the protonated nitro­gen atoms separate the tetra­chlorido­metalate anions, thus reducing electrostatic repulsion between them. Close packing and electrostatic inter­action with anion results in several short C—H⋯Cl contacts (Tables 1 ▸ and 2 ▸).

The layers assemble in the 3D crystal (Fig. 6 ▸) *via* weak inter­molecular forces: the only specific inter-layer contact is C15—H15*B*⋯O1 with C⋯O distances too long to be considered a strong hydrogen bond [3.473 (4) (*a*) and 3.507 (6) Å (*b*)].

## Database survey   

There are three reported dextromethorphan structures deposited in the Cambridge Structural Database (CSD Version 5.37; Groom *et al.*, 2016 ▸). Of these structures, two report structures of the neutral mol­ecule (refcodes XAPTAK and XAPTAK01), one of which (Swamy *et al.*, 2005 ▸) refers to a room-temperature measurement and the other (Scheins *et al.*, 2007 ▸) a high-quality charge-density investigation performed at 20 K.

A protonated form is also known in a form of the bromide salt (refcode DEXORP), in which one solvate water mol­ecule is connected to a protonated nitro­gen atom *via* a hydrogen bond (Gylbert & Carlström, 1977 ▸).

## Synthesis and crystallization   

Dextromethorphan was isolated during the analysis of a proprietary cough syrup using a standard Pharmacopoeia procedure (WHO, 2016 ▸). GC–MS assay of the hexane solution shows dextromethorphan to be a main component, with a small admixture of menthol.

Dextromethorphan was positively identified using NMR and FTIR spectra. Slow evaporation of a hexane solution at 274 K yields crystals which were also identified as dextromethorphan (refcode XAPTAK; Swamy *et al.*, 2005 ▸). Around 20 mg of the solid residue was treated with two drops of concentrated HCl and an excess of cobalt(II) chloride or copper(II) chloride. Overnight standing in a refrigerator yielded crystals of the title compounds. The colors of the resulting solids were characteristic with the tetra­chlorido­cobaltate(II) salt being blue and the tetra­chlorido­cuprate(II) salt yellow. The bright colors of the crystals make them easy to separate from possible crystalline impurities. We expect that levomethorphan would yield similar crystals with the opposite chirality.

**Figure 7:**
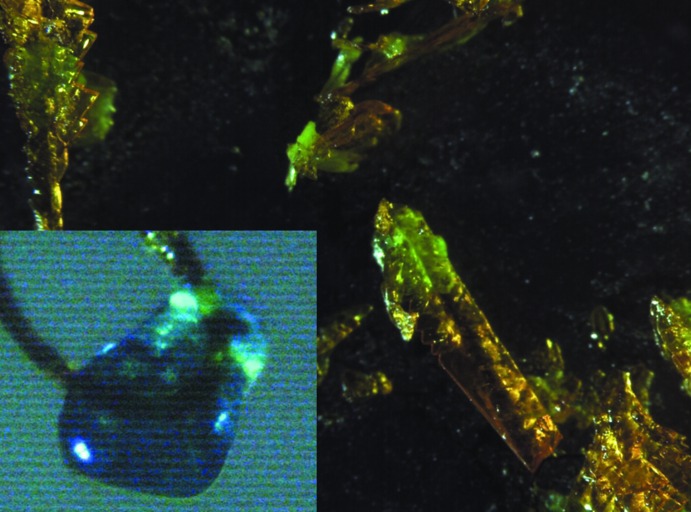
Crystals of the dextromethorphan tetra­chlorido­cobaltate (blue,left corner) and tetra­chlorido­cuprate (yellow) salts. The diagonal image sizes are ∼ 0.6 and 3 mm, respectively.

Crystals suitable for X-ray investigation (Fig. 7 ▸) were cut from larger blocks before mounting on Mitigen loops.

## Refinement   

Crystal data, data collection and structure refinement details are summarized in Table 3 ▸. In (*a*), the hydrogen atom H1 of the protonated amine was refined in an isotropic approximation; idealized methyl groups refined as rotating groups with stretchable bonds and *U*_iso_ = 1.5*U*_iso_(C); all other hydrogen atoms were refined with riding coordinates and stretchable bonds with *U*_iso_ = 1.2*U*_iso_(C). In (*b*), the hydrogen atom were treated in an similar fashion.

## Supplementary Material

Crystal structure: contains datablock(s) a, b. DOI: 10.1107/S2056989016019939/sj5517sup1.cif

Structure factors: contains datablock(s) a. DOI: 10.1107/S2056989016019939/sj5517asup2.hkl

Supporting information file. DOI: 10.1107/S2056989016019939/sj5517asup4.cdx

Structure factors: contains datablock(s) b. DOI: 10.1107/S2056989016019939/sj5517bsup3.hkl

CCDC references: 1522812, 1522811

Additional supporting information:  crystallographic information; 3D view; checkCIF report

## Figures and Tables

**Figure 2:**
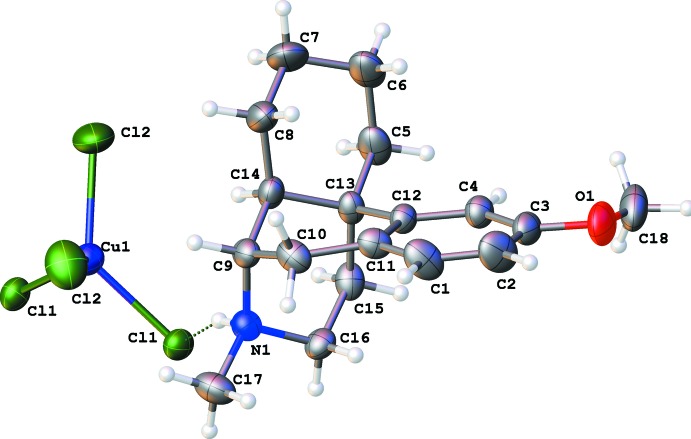
The numbering scheme of the dextromethorphan tetra­chlorido­cuprate complex (*b*) with displacement ellipsoids drawn at the 50% probability level.

**Figure 3:**
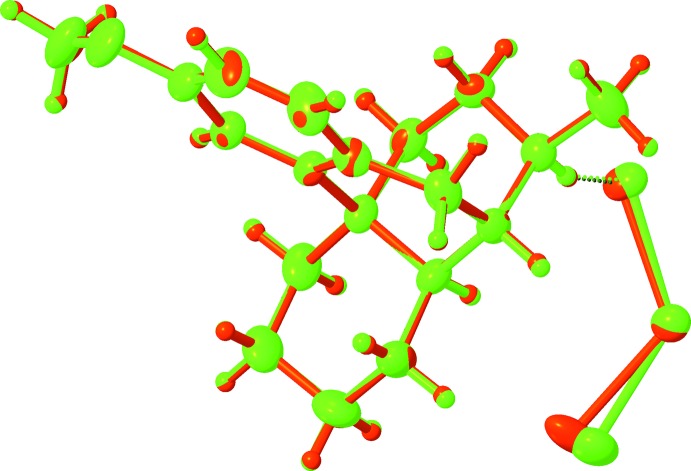
Overlay of the dextromethorphan tetra­chlorido­cobaltate (green) and tetra­chlorido­cuprate (red) complexes.

**Table 1:** Hydrogen-bond geometry (Å, °) for (*a*)

*D*—H⋯*A*	*D*—H	H⋯*A*	*D*⋯*A*	*D*—H⋯*A*
N1—H1⋯Cl1	0.93 (4)	2.26 (4)	3.145 (2)	158 (4)
C10—H10*B*⋯Cl2^i^	0.97 (3)	2.71 (2)	3.609 (3)	153 (1)
C17—H17*B*⋯Cl2^ii^	0.98 (3)	2.75 (3)	3.615 (4)	148 (1)

Symmetry codes: (i) 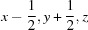; (ii) 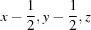.

**Table 2:** Hydrogen-bond geometry (Å, °) for (*b*)

*D*—H⋯*A*	*D*—H	H⋯*A*	*D*⋯*A*	*D*—H⋯*A*
N1—H1⋯Cl1	0.81 (6)	2.46 (6)	3.207 (4)	154 (5)
C10—H10*A*⋯Cl2^i^	0.99	2.83	3.754 (5)	156
C17—H17*B*⋯Cl2^ii^	0.98	2.68	3.590 (5)	156

Symmetry codes: (i) 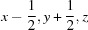; (ii) 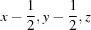.

**Table 3:** Experimental details

	(*a*)	(*b*)
Crystal data		
Chemical formula	(C_18_H_26_NO)_2_[CoCl_4_]	(C_18_H_26_NO)[CuCl_4_]
*M*_r_	745.52	750.13
Crystal system, space group	Monoclinic, *C*2	Monoclinic, *C*2
Temperature (K)	173	173
*a*, *b*, *c* (Å)	13.8447 (6), 9.2316 (4), 14.7018 (6)	13.8066 (16), 9.2934 (12), 14.651 (3)
β (°)	99.605 (2)	99.318 (6)
*V* (Å^3^)	1852.68 (14)	1855.1 (5)
*Z*	2	2
Radiation type	Mo *K*α	Mo *K*α
μ (mm^−1^)	0.79	0.91
Crystal size (mm)	0.48 × 0.26 × 0.14	0.45 × 0.3 × 0.15

Data collection		
Diffractometer	Bruker PHOTON-100 CMOS	Bruker PHOTON-100 CMOS
Absorption correction	Multi-scan (*SADABS*, Bruker, 2015 ▸)	Multi-scan (*SADABS*, Bruker, 2015 ▸)
*T*_min_, *T*_max_	0.761, 0.979	0.714, 0.933
No. of measured, independent and observed [*I* > 2σ(*I*)] reflections	43153, 4803, 4183	31097, 4243, 3521
*R*_int_	0.061	0.047
(sin θ/λ)_max_ (Å^−1^)	0.677	0.649

Refinement		
*R*[*F* ^2^ > 2σ(*F* ^2^)], *wR*(*F* ^2^), *S*	0.034, 0.083, 1.04	0.039, 0.100, 1.08
No. of reflections	4803	4243
No. of parameters	224	209
No. of restraints	1	1
H-atom treatment	H atoms treated by a mixture of independent and constrained refinement	H atoms treated by a mixture of independent and constrained refinement
Δρ_max_, Δρ_min_ (e Å^−3^)	0.50, −0.26	0.51, −0.33
Absolute structure	Flack *x* determined using 1711 quotients [(*I* ^+^)−(*I* ^−^)]/[(*I* ^+^)+(*I* ^−^)] (Parsons *et al.*, 2013 ▸)	Flack *x* determined using 1463 quotients [(*I* ^+^)−(*I* ^−^)]/[(*I* ^+^)+(*I* ^−^)] (Parsons *et al.*, 2013 ▸)
Absolute structure parameter	0.000 (7)	−0.005 (6)

Computer programs: *APEX2* and *SAINT* (Bruker, 2013 ▸), *SHELXT* (Sheldrick, 2015 ▸), *SHELXS97* and *SHELXL97* (Sheldrick, 2008 ▸) and *OLEX2* (Dolomanov *et al.*, 2009 ▸).

## supplementary crystallographic information

### (a) Bis[(9S,13S,14S)-3-methoxy-17-methylmorphinanium] tetrachloridocobaltate Crystal data

**Table:**

(C_18_H_26_NO)_2_[CoCl_4_]	*F*(000) = 786
*M**_r_* = 745.52	*D*_x_ = 1.336 Mg m^−^^3^
Monoclinic, *C*2	Mo *K*α radiation, λ = 0.71073 Å
*a* = 13.8447 (6) Å	Cell parameters from 9174 reflections
*b* = 9.2316 (4) Å	θ = 2.9–28.3°
*c* = 14.7018 (6) Å	µ = 0.79 mm^−^^1^
β = 99.605 (2)°	*T* = 173 K
*V* = 1852.68 (14) Å^3^	Plate, blue
*Z* = 2	0.48 × 0.26 × 0.14 mm

### (a) Bis[(9S,13S,14S)-3-methoxy-17-methylmorphinanium] tetrachloridocobaltate Data collection

**Table:**

Bruker PHOTON-100 CMOS diffractometer	4183 reflections with *I* > 2σ(*I*)
Radiation source: sealed tube	*R*_int_ = 0.061
φ and ω scans	θ_max_ = 28.8°, θ_min_ = 2.8°
Absorption correction: multi-scan (SADABS, Bruker, 2015)	*h* = −18→18
*T*_min_ = 0.761, *T*_max_ = 0.979	*k* = −12→12
43153 measured reflections	*l* = −19→19
4803 independent reflections	

### (a) Bis[(9S,13S,14S)-3-methoxy-17-methylmorphinanium] tetrachloridocobaltate Refinement

**Table:**

Refinement on *F*^2^	Hydrogen site location: mixed
Least-squares matrix: full	H atoms treated by a mixture of independent and constrained refinement
*R*[*F*^2^ > 2σ(*F*^2^)] = 0.034	*w* = 1/[σ^2^(*F*_o_^2^) + (0.0494*P*)^2^ + 0.4319*P*] where *P* = (*F*_o_^2^ + 2*F*_c_^2^)/3
*wR*(*F*^2^) = 0.083	(Δ/σ)_max_ < 0.001
*S* = 1.04	Δρ_max_ = 0.50 e Å^−^^3^
4803 reflections	Δρ_min_ = −0.26 e Å^−^^3^
224 parameters	Absolute structure: Flack *x* determined using 1711 quotients [(*I*^+^)-(*I*^-^)]/[(*I*^+^)+(*I*^-^)] (Parsons et al., 2013)
1 restraint	Absolute structure parameter: 0.000 (7)

### (a) Bis[(9S,13S,14S)-3-methoxy-17-methylmorphinanium] tetrachloridocobaltate Special details

**Table:**

Geometry. All esds (except the esd in the dihedral angle between two l.s. planes) are estimated using the full covariance matrix. The cell esds are taken into account individually in the estimation of esds in distances, angles and torsion angles; correlations between esds in cell parameters are only used when they are defined by crystal symmetry. An approximate (isotropic) treatment of cell esds is used for estimating esds involving l.s. planes.

### (a) Bis[(9S,13S,14S)-3-methoxy-17-methylmorphinanium] tetrachloridocobaltate Fractional atomic coordinates and isotropic or equivalent isotropic displacement parameters (Å^2^)

**Table:**

	*x*	*y*	*z*	*U*_iso_*/*U*_eq_	
O1	0.16362 (18)	0.6052 (3)	0.55317 (15)	0.0489 (6)	
N1	0.25197 (17)	0.4294 (2)	0.12297 (15)	0.0277 (5)	
C3	0.1767 (2)	0.6210 (3)	0.4633 (2)	0.0363 (7)	
C4	0.2614 (2)	0.5804 (3)	0.43052 (18)	0.0302 (5)	
H4	0.316 (2)	0.5398 (15)	0.4727 (15)	0.036*	
C12	0.26809 (18)	0.5975 (3)	0.33709 (17)	0.0257 (5)	
C11	0.1890 (2)	0.6534 (3)	0.27670 (18)	0.0295 (6)	
C1	0.1055 (2)	0.6958 (4)	0.3114 (2)	0.0429 (7)	
H1A	0.055 (2)	0.7347 (18)	0.2728 (17)	0.051*	
C2	0.0990 (2)	0.6796 (5)	0.4031 (2)	0.0467 (8)	
H2	0.037 (3)	0.7107 (14)	0.4268 (10)	0.056*	
C18	0.2367 (3)	0.5315 (4)	0.6139 (2)	0.0544 (10)	
H18A	0.2440 (18)	0.436 (3)	0.5914 (14)	0.082*	
H18B	0.2184 (13)	0.526 (3)	0.6733 (18)	0.082*	
H18C	0.2970 (18)	0.582 (2)	0.6180 (18)	0.082*	
C10	0.1886 (2)	0.6676 (3)	0.17396 (18)	0.0320 (6)	
H10A	0.1270 (16)	0.6305 (10)	0.1415 (9)	0.038*	
H10B	0.1917 (2)	0.770 (3)	0.1590 (4)	0.038*	
C9	0.2708 (2)	0.5904 (3)	0.13877 (18)	0.0277 (5)	
H9	0.2785 (4)	0.6312 (16)	0.084 (2)	0.033*	
C14	0.3669 (2)	0.6068 (3)	0.20608 (18)	0.0291 (5)	
H14	0.4161 (19)	0.554 (2)	0.1814 (9)	0.035*	
C8	0.3998 (3)	0.7643 (4)	0.2153 (2)	0.0424 (7)	
H8A	0.3525 (15)	0.8185 (17)	0.2353 (6)	0.051*	
H8B	0.4083 (3)	0.7987 (12)	0.1591 (17)	0.051*	
C7	0.4956 (3)	0.7790 (5)	0.2833 (2)	0.0519 (9)	
H7A	0.5481 (18)	0.7293 (17)	0.2587 (9)	0.062*	
H7B	0.5132 (7)	0.881 (3)	0.2911 (3)	0.062*	
C6	0.4853 (2)	0.7140 (5)	0.3763 (2)	0.0458 (8)	
H6A	0.4353 (13)	0.7693 (15)	0.4036 (8)	0.055*	
H6B	0.5490 (17)	0.7216 (5)	0.4192 (12)	0.055*	
C5	0.4548 (2)	0.5556 (4)	0.36542 (19)	0.0374 (7)	
H5A	0.4490 (2)	0.5175 (12)	0.4233 (17)	0.045*	
H5B	0.5036 (14)	0.5032 (16)	0.3426 (6)	0.045*	
C13	0.35654 (19)	0.5369 (3)	0.29939 (17)	0.0267 (5)	
C15	0.33688 (19)	0.3737 (4)	0.28147 (16)	0.0297 (5)	
H15A	0.3903 (14)	0.3325 (11)	0.2615 (5)	0.036*	
H15B	0.3292 (3)	0.3292 (12)	0.3359 (14)	0.036*	
C16	0.2462 (2)	0.3475 (3)	0.20999 (18)	0.0302 (6)	
H16A	0.2401 (3)	0.248 (3)	0.1968 (4)	0.036*	
H16B	0.1903 (15)	0.3772 (8)	0.2337 (7)	0.036*	
C17	0.1662 (2)	0.3993 (4)	0.0495 (2)	0.0412 (7)	
H17A	0.1063 (15)	0.427 (3)	0.0712 (9)	0.062*	
H17B	0.1640 (12)	0.296 (3)	0.0350 (13)	0.062*	
H17C	0.1725 (10)	0.455 (3)	−0.0055 (16)	0.062*	
Co1	0.5000	0.37707 (5)	0.0000	0.02972 (14)	
Cl1	0.41931 (5)	0.22074 (7)	0.08249 (4)	0.03266 (16)	
Cl2	0.61476 (7)	0.52069 (9)	0.08226 (7)	0.0570 (3)	
H1	0.306 (3)	0.389 (5)	0.102 (2)	0.045 (9)*	

### (a) Bis[(9S,13S,14S)-3-methoxy-17-methylmorphinanium] tetrachloridocobaltate Atomic displacement parameters (Å^2^)

**Table:**

	*U*^11^	*U*^22^	*U*^33^	*U*^12^	*U*^13^	*U*^23^
O1	0.0563 (15)	0.0602 (15)	0.0369 (11)	0.0015 (13)	0.0271 (10)	−0.0068 (11)
N1	0.0275 (11)	0.0271 (11)	0.0289 (11)	0.0014 (9)	0.0057 (9)	−0.0015 (8)
C3	0.0418 (16)	0.0377 (16)	0.0331 (14)	−0.0024 (13)	0.0173 (12)	−0.0085 (12)
C4	0.0307 (13)	0.0330 (14)	0.0279 (12)	0.0019 (12)	0.0083 (10)	−0.0013 (11)
C12	0.0254 (12)	0.0264 (12)	0.0267 (11)	0.0021 (11)	0.0079 (9)	−0.0019 (10)
C11	0.0274 (13)	0.0308 (14)	0.0306 (13)	0.0074 (11)	0.0054 (10)	−0.0041 (10)
C1	0.0319 (15)	0.0512 (19)	0.0450 (16)	0.0152 (14)	0.0048 (13)	−0.0101 (15)
C2	0.0362 (16)	0.060 (2)	0.0474 (17)	0.0076 (15)	0.0187 (14)	−0.0163 (16)
C18	0.083 (3)	0.049 (2)	0.0373 (17)	0.000 (2)	0.0278 (18)	0.0019 (15)
C10	0.0346 (14)	0.0312 (13)	0.0286 (12)	0.0069 (12)	0.0009 (11)	0.0000 (10)
C9	0.0344 (14)	0.0254 (12)	0.0236 (11)	−0.0031 (11)	0.0054 (10)	0.0037 (10)
C14	0.0279 (13)	0.0341 (14)	0.0271 (12)	−0.0018 (11)	0.0097 (10)	0.0007 (11)
C8	0.0501 (19)	0.0430 (18)	0.0353 (15)	−0.0152 (15)	0.0104 (13)	−0.0005 (13)
C7	0.0452 (19)	0.062 (2)	0.0488 (19)	−0.0242 (17)	0.0089 (15)	−0.0114 (17)
C6	0.0308 (15)	0.065 (2)	0.0392 (15)	−0.0089 (16)	−0.0003 (12)	−0.0110 (16)
C5	0.0239 (13)	0.057 (2)	0.0296 (13)	0.0048 (13)	0.0008 (11)	−0.0016 (13)
C13	0.0215 (12)	0.0371 (15)	0.0220 (11)	0.0024 (11)	0.0053 (9)	0.0004 (10)
C15	0.0332 (13)	0.0313 (13)	0.0259 (11)	0.0103 (13)	0.0089 (10)	0.0048 (12)
C16	0.0345 (14)	0.0242 (14)	0.0335 (13)	−0.0016 (11)	0.0108 (11)	0.0024 (10)
C17	0.0424 (16)	0.0376 (18)	0.0391 (15)	−0.0017 (14)	−0.0062 (12)	−0.0043 (13)
Co1	0.0263 (3)	0.0280 (3)	0.0351 (3)	0.000	0.0058 (2)	0.000
Cl1	0.0312 (3)	0.0354 (3)	0.0342 (3)	−0.0025 (3)	0.0137 (3)	−0.0038 (3)
Cl2	0.0577 (5)	0.0342 (4)	0.0711 (6)	−0.0164 (4)	−0.0130 (4)	0.0022 (4)

### (a) Bis[(9S,13S,14S)-3-methoxy-17-methylmorphinanium] tetrachloridocobaltate Geometric parameters (Å, º)

**Table:**

O1—C3	1.372 (3)	C14—C13	1.544 (4)
O1—C18	1.408 (5)	C8—H8A	0.91 (3)
N1—C9	1.520 (3)	C8—H8B	0.91 (3)
N1—C16	1.500 (3)	C8—C7	1.527 (5)
N1—C17	1.491 (3)	C7—H7A	0.98 (3)
N1—H1	0.93 (4)	C7—H7B	0.98 (3)
C3—C4	1.391 (4)	C7—C6	1.521 (5)
C3—C2	1.384 (5)	C6—H6A	1.00 (3)
C4—H4	0.97 (3)	C6—H6B	1.00 (3)
C4—C12	1.401 (3)	C6—C5	1.523 (6)
C12—C11	1.389 (4)	C5—H5A	0.94 (3)
C12—C13	1.532 (3)	C5—H5B	0.94 (3)
C11—C1	1.396 (4)	C5—C13	1.543 (4)
C11—C10	1.515 (4)	C13—C15	1.546 (4)
C1—H1A	0.90 (4)	C15—H15A	0.92 (2)
C1—C2	1.375 (5)	C15—H15B	0.92 (2)
C2—H2	1.02 (4)	C15—C16	1.516 (4)
C18—H18A	0.95 (3)	C16—H16A	0.94 (2)
C18—H18B	0.95 (3)	C16—H16B	0.94 (2)
C18—H18C	0.95 (3)	C17—H17A	0.97 (2)
C10—H10A	0.97 (2)	C17—H17B	0.97 (2)
C10—H10B	0.97 (2)	C17—H17C	0.97 (2)
C10—C9	1.506 (4)	Co1—Cl1^i^	2.2920 (7)
C9—H9	0.91 (3)	Co1—Cl1	2.2921 (7)
C9—C14	1.527 (4)	Co1—Cl2^i^	2.2592 (8)
C14—H14	0.96 (4)	Co1—Cl2	2.2591 (8)
C14—C8	1.523 (4)		
			
C3—O1—C18	117.8 (3)	H8A—C8—H8B	108.0
C9—N1—H1	109 (3)	C7—C8—H8A	109.5
C16—N1—C9	113.3 (2)	C7—C8—H8B	109.5
C16—N1—H1	104 (2)	C8—C7—H7A	109.5
C17—N1—C9	112.9 (2)	C8—C7—H7B	109.5
C17—N1—C16	112.0 (2)	H7A—C7—H7B	108.1
C17—N1—H1	106 (2)	C6—C7—C8	110.6 (3)
O1—C3—C4	124.0 (3)	C6—C7—H7A	109.5
O1—C3—C2	116.3 (3)	C6—C7—H7B	109.5
C2—C3—C4	119.7 (3)	C7—C6—H6A	109.6
C3—C4—H4	119.8	C7—C6—H6B	109.6
C3—C4—C12	120.4 (3)	C7—C6—C5	110.5 (3)
C12—C4—H4	119.8	H6A—C6—H6B	108.1
C4—C12—C13	120.1 (2)	C5—C6—H6A	109.6
C11—C12—C4	119.6 (2)	C5—C6—H6B	109.6
C11—C12—C13	119.8 (2)	C6—C5—H5A	109.2
C12—C11—C1	118.9 (3)	C6—C5—H5B	109.2
C12—C11—C10	122.7 (2)	C6—C5—C13	111.9 (3)
C1—C11—C10	118.3 (3)	H5A—C5—H5B	107.9
C11—C1—H1A	119.3	C13—C5—H5A	109.2
C2—C1—C11	121.4 (3)	C13—C5—H5B	109.2
C2—C1—H1A	119.3	C12—C13—C14	111.5 (2)
C3—C2—H2	120.1	C12—C13—C5	113.9 (2)
C1—C2—C3	119.9 (3)	C12—C13—C15	106.8 (2)
C1—C2—H2	120.1	C14—C13—C15	107.2 (2)
O1—C18—H18A	109.5	C5—C13—C14	108.0 (2)
O1—C18—H18B	109.5	C5—C13—C15	109.1 (2)
O1—C18—H18C	109.5	C13—C15—H15A	109.2
H18A—C18—H18B	109.5	C13—C15—H15B	109.2
H18A—C18—H18C	109.5	H15A—C15—H15B	107.9
H18B—C18—H18C	109.5	C16—C15—C13	112.1 (2)
C11—C10—H10A	108.5	C16—C15—H15A	109.2
C11—C10—H10B	108.5	C16—C15—H15B	109.2
H10A—C10—H10B	107.5	N1—C16—C15	110.8 (2)
C9—C10—C11	115.1 (2)	N1—C16—H16A	109.5
C9—C10—H10A	108.5	N1—C16—H16B	109.5
C9—C10—H10B	108.5	C15—C16—H16A	109.5
N1—C9—H9	108.3	C15—C16—H16B	109.5
N1—C9—C14	107.7 (2)	H16A—C16—H16B	108.1
C10—C9—N1	113.3 (2)	N1—C17—H17A	109.5
C10—C9—H9	108.3	N1—C17—H17B	109.5
C10—C9—C14	110.9 (2)	N1—C17—H17C	109.5
C14—C9—H9	108.3	H17A—C17—H17B	109.5
C9—C14—H14	107.5	H17A—C17—H17C	109.5
C9—C14—C13	109.5 (2)	H17B—C17—H17C	109.5
C8—C14—C9	111.5 (2)	Cl1^i^—Co1—Cl1	101.95 (4)
C8—C14—H14	107.5	Cl2^i^—Co1—Cl1	106.94 (3)
C8—C14—C13	113.0 (2)	Cl2—Co1—Cl1^i^	106.94 (3)
C13—C14—H14	107.5	Cl2—Co1—Cl1	116.59 (3)
C14—C8—H8A	109.5	Cl2^i^—Co1—Cl1^i^	116.59 (3)
C14—C8—H8B	109.5	Cl2—Co1—Cl2^i^	108.13 (5)
C14—C8—C7	110.9 (3)		
			
O1—C3—C4—C12	178.7 (3)	C10—C9—C14—C13	61.9 (3)
O1—C3—C2—C1	−178.5 (3)	C9—N1—C16—C15	−54.1 (3)
N1—C9—C14—C8	171.6 (2)	C9—C14—C8—C7	179.6 (2)
N1—C9—C14—C13	−62.6 (3)	C9—C14—C13—C12	−54.0 (3)
C3—C4—C12—C11	−1.0 (4)	C9—C14—C13—C5	−180.0 (2)
C3—C4—C12—C13	−173.0 (3)	C9—C14—C13—C15	62.5 (3)
C4—C3—C2—C1	0.7 (5)	C14—C8—C7—C6	−55.7 (4)
C4—C12—C11—C1	2.2 (4)	C14—C13—C15—C16	−57.8 (3)
C4—C12—C11—C10	−176.6 (3)	C8—C14—C13—C12	71.0 (3)
C4—C12—C13—C14	−162.0 (2)	C8—C14—C13—C5	−55.0 (3)
C4—C12—C13—C5	−39.4 (4)	C8—C14—C13—C15	−172.5 (2)
C4—C12—C13—C15	81.2 (3)	C8—C7—C6—C5	57.4 (4)
C12—C11—C1—C2	−1.9 (5)	C7—C6—C5—C13	−59.0 (4)
C12—C11—C10—C9	11.7 (4)	C6—C5—C13—C12	−68.1 (3)
C12—C13—C15—C16	61.8 (3)	C6—C5—C13—C14	56.4 (3)
C11—C12—C13—C14	26.0 (4)	C6—C5—C13—C15	172.6 (2)
C11—C12—C13—C5	148.6 (3)	C5—C13—C15—C16	−174.6 (2)
C11—C12—C13—C15	−90.8 (3)	C13—C12—C11—C1	174.2 (3)
C11—C1—C2—C3	0.4 (6)	C13—C12—C11—C10	−4.6 (4)
C11—C10—C9—N1	81.1 (3)	C13—C14—C8—C7	55.7 (3)
C11—C10—C9—C14	−40.1 (3)	C13—C15—C16—N1	53.7 (3)
C1—C11—C10—C9	−167.1 (3)	C16—N1—C9—C10	−64.7 (3)
C2—C3—C4—C12	−0.4 (5)	C16—N1—C9—C14	58.4 (3)
C18—O1—C3—C4	−6.0 (5)	C17—N1—C9—C10	63.9 (3)
C18—O1—C3—C2	173.2 (3)	C17—N1—C9—C14	−173.0 (2)
C10—C11—C1—C2	176.9 (3)	C17—N1—C16—C15	176.9 (2)
C10—C9—C14—C8	−63.9 (3)		

Symmetry code: (i) −*x*+1, *y*, −*z*.

### (a) Bis[(9S,13S,14S)-3-methoxy-17-methylmorphinanium] tetrachloridocobaltate Hydrogen-bond geometry (Å, º)

**Table:**

*D*—H···*A*	*D*—H	H···*A*	*D*···*A*	*D*—H···*A*
N1—H1···Cl1	0.93 (4)	2.26 (4)	3.145 (2)	158 (4)
C10—H10*B*···Cl2^ii^	0.97 (3)	2.71 (2)	3.609 (3)	153 (1)
C17—H17*B*···Cl2^iii^	0.98 (3)	2.75 (3)	3.615 (4)	148 (1)

Symmetry codes: (ii) *x*−1/2, *y*+1/2, *z*; (iii) *x*−1/2, *y*−1/2, *z*.

### (b) Bis[(9S,13S,14S)-3-methoxy-17-methylmorphinanium] tetrachloridocuprate Crystal data

**Table:**

(C_18_H_26_NO)[CuCl_4_]	*F*(000) = 790
*M**_r_* = 750.13	*D*_x_ = 1.343 Mg m^−^^3^
Monoclinic, *C*2	Mo *K*α radiation, λ = 0.71073 Å
*a* = 13.8066 (16) Å	Cell parameters from 1064 reflections
*b* = 9.2934 (12) Å	θ = 3.1–24.8°
*c* = 14.651 (3) Å	µ = 0.91 mm^−^^1^
β = 99.318 (6)°	*T* = 173 K
*V* = 1855.1 (5) Å^3^	Plate, yellow
*Z* = 2	0.45 × 0.3 × 0.15 mm

### (b) Bis[(9S,13S,14S)-3-methoxy-17-methylmorphinanium] tetrachloridocuprate Data collection

**Table:**

Bruker PHOTON-100 CMOS diffractometer	3521 reflections with *I* > 2σ(*I*)
Radiation source: sealedtube	*R*_int_ = 0.047
φ and ω scans	θ_max_ = 27.5°, θ_min_ = 2.8°
Absorption correction: multi-scan (SADABS, Bruker, 2015)	*h* = −17→17
*T*_min_ = 0.714, *T*_max_ = 0.933	*k* = −12→12
31097 measured reflections	*l* = −19→19
4243 independent reflections	

### (b) Bis[(9S,13S,14S)-3-methoxy-17-methylmorphinanium] tetrachloridocuprate Refinement

**Table:**

Refinement on *F*^2^	Hydrogen site location: mixed
Least-squares matrix: full	H atoms treated by a mixture of independent and constrained refinement
*R*[*F*^2^ > 2σ(*F*^2^)] = 0.039	*w* = 1/[σ^2^(*F*_o_^2^) + (0.0512*P*)^2^ + 1.3109*P*] where *P* = (*F*_o_^2^ + 2*F*_c_^2^)/3
*wR*(*F*^2^) = 0.100	(Δ/σ)_max_ < 0.001
*S* = 1.08	Δρ_max_ = 0.51 e Å^−^^3^
4243 reflections	Δρ_min_ = −0.33 e Å^−^^3^
209 parameters	Absolute structure: Flack *x* determined using 1463 quotients [(*I*^+^)-(*I*^-^)]/[(*I*^+^)+(*I*^-^)] (Parsons et al., 2013)
1 restraint	Absolute structure parameter: −0.005 (6)

### (b) Bis[(9S,13S,14S)-3-methoxy-17-methylmorphinanium] tetrachloridocuprate Special details

**Table:**

Geometry. All esds (except the esd in the dihedral angle between two l.s. planes) are estimated using the full covariance matrix. The cell esds are taken into account individually in the estimation of esds in distances, angles and torsion angles; correlations between esds in cell parameters are only used when they are defined by crystal symmetry. An approximate (isotropic) treatment of cell esds is used for estimating esds involving l.s. planes.

### (b) Bis[(9S,13S,14S)-3-methoxy-17-methylmorphinanium] tetrachloridocuprate Fractional atomic coordinates and isotropic or equivalent isotropic displacement parameters (Å^2^)

**Table:**

	*x*	*y*	*z*	*U*_iso_*/*U*_eq_	
O1	0.1671 (3)	0.6033 (4)	0.5575 (2)	0.0560 (9)	
N1	0.2499 (3)	0.4329 (4)	0.1218 (2)	0.0329 (8)	
H1	0.296 (4)	0.402 (6)	0.100 (4)	0.049*	
C1	0.1031 (3)	0.6887 (6)	0.3155 (4)	0.0497 (12)	
H1A	0.0477	0.7261	0.2756	0.060*	
C2	0.0989 (4)	0.6737 (7)	0.4073 (4)	0.0535 (13)	
H2	0.0413	0.7018	0.4304	0.064*	
C3	0.1774 (3)	0.6181 (5)	0.4669 (3)	0.0400 (10)	
C4	0.2621 (3)	0.5790 (5)	0.4319 (3)	0.0340 (9)	
H4	0.3167	0.5404	0.4723	0.041*	
C5	0.4546 (3)	0.5603 (6)	0.3624 (3)	0.0456 (12)	
H5A	0.4499	0.5191	0.4239	0.055*	
H5B	0.5064	0.5072	0.3369	0.055*	
C6	0.4833 (4)	0.7196 (7)	0.3735 (3)	0.0562 (14)	
H6A	0.4333	0.7725	0.4016	0.067*	
H6B	0.5471	0.7288	0.4150	0.067*	
C7	0.4911 (4)	0.7841 (7)	0.2798 (4)	0.0639 (16)	
H7A	0.5438	0.7347	0.2535	0.077*	
H7B	0.5085	0.8872	0.2873	0.077*	
C8	0.3941 (4)	0.7686 (6)	0.2138 (3)	0.0506 (13)	
H8A	0.3426	0.8246	0.2376	0.061*	
H8B	0.4014	0.8078	0.1524	0.061*	
C9	0.2665 (3)	0.5938 (4)	0.1385 (3)	0.0325 (9)	
H9	0.2729	0.6402	0.0781	0.039*	
C10	0.1834 (3)	0.6653 (5)	0.1759 (3)	0.0374 (9)	
H10A	0.1834	0.7690	0.1604	0.045*	
H10B	0.1208	0.6242	0.1441	0.045*	
C11	0.1865 (3)	0.6506 (4)	0.2785 (3)	0.0335 (9)	
C12	0.2669 (3)	0.5965 (4)	0.3383 (3)	0.0287 (8)	
C13	0.3559 (3)	0.5401 (5)	0.2980 (3)	0.0313 (9)	
C14	0.3634 (3)	0.6116 (5)	0.2038 (3)	0.0340 (9)	
H14	0.4149	0.5590	0.1763	0.041*	
C15	0.3388 (3)	0.3775 (5)	0.2790 (3)	0.0364 (9)	
H15A	0.3966	0.3365	0.2562	0.044*	
H15B	0.3322	0.3283	0.3376	0.044*	
C16	0.2482 (3)	0.3492 (5)	0.2089 (3)	0.0349 (9)	
H16A	0.2440	0.2451	0.1943	0.042*	
H16B	0.1893	0.3761	0.2356	0.042*	
C17	0.1634 (4)	0.4004 (5)	0.0501 (3)	0.0492 (12)	
H17A	0.1031	0.4261	0.0736	0.074*	
H17B	0.1625	0.2975	0.0355	0.074*	
H17C	0.1676	0.4562	−0.0058	0.074*	
C18	0.2410 (5)	0.5329 (7)	0.6180 (4)	0.0624 (16)	
H18A	0.2501	0.4356	0.5950	0.094*	
H18B	0.2222	0.5270	0.6796	0.094*	
H18C	0.3026	0.5868	0.6218	0.094*	
Cu1	0.5000	0.36997 (7)	0.0000	0.0369 (2)	
Cl1	0.41472 (7)	0.21415 (11)	0.07511 (7)	0.0354 (2)	
Cl2	0.62112 (11)	0.51975 (14)	0.05807 (10)	0.0632 (4)	

### (b) Bis[(9S,13S,14S)-3-methoxy-17-methylmorphinanium] tetrachloridocuprate Atomic displacement parameters (Å^2^)

**Table:**

	*U*^11^	*U*^22^	*U*^33^	*U*^12^	*U*^13^	*U*^23^
O1	0.068 (2)	0.066 (2)	0.043 (2)	0.0020 (19)	0.0321 (18)	−0.0075 (17)
N1	0.0336 (19)	0.0322 (17)	0.0332 (19)	0.0029 (15)	0.0062 (15)	−0.0022 (15)
C1	0.036 (2)	0.059 (3)	0.053 (3)	0.017 (2)	0.005 (2)	−0.014 (2)
C2	0.036 (2)	0.067 (3)	0.061 (3)	0.010 (2)	0.020 (2)	−0.016 (3)
C3	0.047 (3)	0.040 (2)	0.038 (2)	−0.003 (2)	0.020 (2)	−0.0082 (19)
C4	0.036 (2)	0.035 (2)	0.033 (2)	0.0031 (17)	0.0112 (17)	−0.0017 (17)
C5	0.027 (2)	0.073 (4)	0.036 (2)	0.004 (2)	−0.0010 (17)	−0.002 (2)
C6	0.036 (2)	0.087 (4)	0.044 (3)	−0.013 (3)	0.000 (2)	−0.011 (3)
C7	0.052 (3)	0.075 (4)	0.064 (4)	−0.034 (3)	0.011 (3)	−0.012 (3)
C8	0.059 (3)	0.055 (3)	0.038 (3)	−0.023 (2)	0.008 (2)	0.001 (2)
C9	0.039 (2)	0.031 (2)	0.027 (2)	−0.0030 (17)	0.0024 (16)	0.0040 (16)
C10	0.039 (2)	0.034 (2)	0.036 (2)	0.0091 (18)	−0.0027 (18)	−0.0012 (18)
C11	0.027 (2)	0.032 (2)	0.042 (2)	0.0062 (16)	0.0047 (17)	−0.0049 (18)
C12	0.0298 (19)	0.0282 (19)	0.030 (2)	0.0030 (16)	0.0097 (16)	−0.0006 (16)
C13	0.0249 (19)	0.043 (2)	0.027 (2)	0.0045 (17)	0.0049 (15)	0.0007 (18)
C14	0.033 (2)	0.041 (2)	0.029 (2)	−0.0017 (18)	0.0101 (17)	0.0025 (18)
C15	0.041 (2)	0.036 (2)	0.034 (2)	0.015 (2)	0.0109 (16)	0.004 (2)
C16	0.039 (2)	0.028 (2)	0.039 (2)	0.0003 (17)	0.0110 (17)	0.0042 (18)
C17	0.052 (3)	0.041 (3)	0.049 (3)	−0.002 (2)	−0.008 (2)	−0.008 (2)
C18	0.099 (5)	0.054 (3)	0.041 (3)	0.002 (3)	0.033 (3)	0.006 (3)
Cu1	0.0381 (4)	0.0337 (4)	0.0402 (4)	0.000	0.0105 (3)	0.000
Cl1	0.0329 (5)	0.0376 (5)	0.0381 (5)	−0.0018 (4)	0.0126 (4)	−0.0028 (4)
Cl2	0.0780 (10)	0.0484 (7)	0.0639 (8)	−0.0284 (7)	0.0131 (7)	−0.0041 (6)

### (b) Bis[(9S,13S,14S)-3-methoxy-17-methylmorphinanium] tetrachloridocuprate Geometric parameters (Å, º)

**Table:**

O1—C3	1.366 (5)	C9—H9	1.0000
O1—C18	1.401 (7)	C9—C10	1.504 (6)
N1—H1	0.81 (5)	C9—C14	1.523 (6)
N1—C9	1.526 (5)	C10—H10A	0.9900
N1—C16	1.499 (5)	C10—H10B	0.9900
N1—C17	1.487 (6)	C10—C11	1.503 (6)
C1—H1A	0.9500	C11—C12	1.392 (6)
C1—C2	1.363 (7)	C12—C13	1.539 (5)
C1—C11	1.395 (6)	C13—C14	1.551 (6)
C2—H2	0.9500	C13—C15	1.548 (7)
C2—C3	1.377 (7)	C14—H14	1.0000
C3—C4	1.398 (6)	C15—H15A	0.9900
C4—H4	0.9500	C15—H15B	0.9900
C4—C12	1.394 (6)	C15—C16	1.507 (6)
C5—H5A	0.9900	C16—H16A	0.9900
C5—H5B	0.9900	C16—H16B	0.9900
C5—C6	1.535 (9)	C17—H17A	0.9800
C5—C13	1.538 (6)	C17—H17B	0.9800
C6—H6A	0.9900	C17—H17C	0.9800
C6—H6B	0.9900	C18—H18A	0.9800
C6—C7	1.517 (8)	C18—H18B	0.9800
C7—H7A	0.9900	C18—H18C	0.9800
C7—H7B	0.9900	Cu1—Cl1^i^	2.2615 (11)
C7—C8	1.526 (7)	Cu1—Cl1	2.2614 (11)
C8—H8A	0.9900	Cu1—Cl2	2.2354 (13)
C8—H8B	0.9900	Cu1—Cl2^i^	2.2354 (13)
C8—C14	1.519 (6)		
			
C3—O1—C18	118.8 (4)	C11—C10—C9	115.1 (3)
C9—N1—H1	108 (4)	C11—C10—H10A	108.5
C16—N1—H1	106 (4)	C11—C10—H10B	108.5
C16—N1—C9	113.3 (3)	C1—C11—C10	118.2 (4)
C17—N1—H1	104 (4)	C12—C11—C1	118.1 (4)
C17—N1—C9	113.3 (3)	C12—C11—C10	123.6 (4)
C17—N1—C16	112.0 (3)	C4—C12—C13	120.4 (3)
C2—C1—H1A	119.1	C11—C12—C4	120.1 (4)
C2—C1—C11	121.8 (4)	C11—C12—C13	119.1 (4)
C11—C1—H1A	119.1	C5—C13—C12	114.0 (3)
C1—C2—H2	119.7	C5—C13—C14	108.3 (3)
C1—C2—C3	120.6 (4)	C5—C13—C15	108.9 (4)
C3—C2—H2	119.7	C12—C13—C14	111.6 (3)
O1—C3—C2	117.3 (4)	C12—C13—C15	107.0 (3)
O1—C3—C4	123.7 (4)	C15—C13—C14	106.8 (3)
C2—C3—C4	119.0 (4)	C8—C14—C9	111.6 (4)
C3—C4—H4	119.8	C8—C14—C13	112.5 (4)
C12—C4—C3	120.4 (4)	C8—C14—H14	107.7
C12—C4—H4	119.8	C9—C14—C13	109.5 (3)
H5A—C5—H5B	107.9	C9—C14—H14	107.7
C6—C5—H5A	109.3	C13—C14—H14	107.7
C6—C5—H5B	109.3	C13—C15—H15A	109.1
C6—C5—C13	111.7 (4)	C13—C15—H15B	109.1
C13—C5—H5A	109.3	H15A—C15—H15B	107.9
C13—C5—H5B	109.3	C16—C15—C13	112.3 (3)
C5—C6—H6A	109.7	C16—C15—H15A	109.1
C5—C6—H6B	109.7	C16—C15—H15B	109.1
H6A—C6—H6B	108.2	N1—C16—C15	111.4 (3)
C7—C6—C5	109.9 (4)	N1—C16—H16A	109.4
C7—C6—H6A	109.7	N1—C16—H16B	109.4
C7—C6—H6B	109.7	C15—C16—H16A	109.4
C6—C7—H7A	109.5	C15—C16—H16B	109.4
C6—C7—H7B	109.5	H16A—C16—H16B	108.0
C6—C7—C8	110.7 (4)	N1—C17—H17A	109.5
H7A—C7—H7B	108.1	N1—C17—H17B	109.5
C8—C7—H7A	109.5	N1—C17—H17C	109.5
C8—C7—H7B	109.5	H17A—C17—H17B	109.5
C7—C8—H8A	109.5	H17A—C17—H17C	109.5
C7—C8—H8B	109.5	H17B—C17—H17C	109.5
H8A—C8—H8B	108.1	O1—C18—H18A	109.5
C14—C8—C7	110.8 (5)	O1—C18—H18B	109.5
C14—C8—H8A	109.5	O1—C18—H18C	109.5
C14—C8—H8B	109.5	H18A—C18—H18B	109.5
N1—C9—H9	108.3	H18A—C18—H18C	109.5
C10—C9—N1	112.8 (3)	H18B—C18—H18C	109.5
C10—C9—H9	108.3	Cl1—Cu1—Cl1^i^	100.36 (6)
C10—C9—C14	111.6 (3)	Cl2—Cu1—Cl1^i^	99.65 (5)
C14—C9—N1	107.5 (3)	Cl2^i^—Cu1—Cl1	99.65 (5)
C14—C9—H9	108.3	Cl2^i^—Cu1—Cl1^i^	129.04 (4)
C9—C10—H10A	108.5	Cl2—Cu1—Cl1	129.04 (4)
C9—C10—H10B	108.5	Cl2^i^—Cu1—Cl2	102.97 (9)
H10A—C10—H10B	107.5		
			
O1—C3—C4—C12	179.5 (4)	C9—C10—C11—C1	−167.6 (4)
N1—C9—C10—C11	82.2 (4)	C9—C10—C11—C12	10.2 (6)
N1—C9—C14—C8	171.7 (3)	C10—C9—C14—C8	−64.1 (5)
N1—C9—C14—C13	−63.0 (4)	C10—C9—C14—C13	61.2 (5)
C1—C2—C3—O1	−178.6 (5)	C10—C11—C12—C4	−176.6 (4)
C1—C2—C3—C4	0.9 (8)	C10—C11—C12—C13	−3.6 (6)
C1—C11—C12—C4	1.2 (6)	C11—C1—C2—C3	−0.8 (9)
C1—C11—C12—C13	174.2 (4)	C11—C12—C13—C5	148.6 (4)
C2—C1—C11—C10	177.7 (5)	C11—C12—C13—C14	25.5 (5)
C2—C1—C11—C12	−0.2 (7)	C11—C12—C13—C15	−91.0 (4)
C2—C3—C4—C12	0.1 (7)	C12—C13—C14—C8	71.2 (5)
C3—C4—C12—C11	−1.2 (6)	C12—C13—C14—C9	−53.6 (5)
C3—C4—C12—C13	−174.1 (4)	C12—C13—C15—C16	61.9 (4)
C4—C12—C13—C5	−38.4 (6)	C13—C5—C6—C7	−59.2 (6)
C4—C12—C13—C14	−161.5 (4)	C13—C15—C16—N1	53.3 (4)
C4—C12—C13—C15	82.0 (4)	C14—C9—C10—C11	−39.0 (5)
C5—C6—C7—C8	58.2 (6)	C14—C13—C15—C16	−57.7 (4)
C5—C13—C14—C8	−55.1 (5)	C15—C13—C14—C8	−172.2 (4)
C5—C13—C14—C9	−179.8 (4)	C15—C13—C14—C9	63.1 (4)
C5—C13—C15—C16	−174.4 (3)	C16—N1—C9—C10	−65.5 (4)
C6—C5—C13—C12	−68.5 (5)	C16—N1—C9—C14	58.0 (4)
C6—C5—C13—C14	56.3 (5)	C17—N1—C9—C10	63.5 (5)
C6—C5—C13—C15	172.1 (4)	C17—N1—C9—C14	−173.1 (3)
C6—C7—C8—C14	−57.0 (6)	C17—N1—C16—C15	177.1 (4)
C7—C8—C14—C9	179.7 (4)	C18—O1—C3—C2	173.1 (5)
C7—C8—C14—C13	56.1 (5)	C18—O1—C3—C4	−6.3 (7)
C9—N1—C16—C15	−53.3 (5)		

Symmetry code: (i) −*x*+1, *y*, −*z*.

### (b) Bis[(9S,13S,14S)-3-methoxy-17-methylmorphinanium] tetrachloridocuprate Hydrogen-bond geometry (Å, º)

**Table:**

*D*—H···*A*	*D*—H	H···*A*	*D*···*A*	*D*—H···*A*
N1—H1···Cl1	0.81 (6)	2.46 (6)	3.207 (4)	154 (5)
C10—H10*A*···Cl2^ii^	0.99	2.83	3.754 (5)	156
C17—H17*B*···Cl2^iii^	0.98	2.68	3.590 (5)	156

Symmetry codes: (ii) *x*−1/2, *y*+1/2, *z*; (iii) *x*−1/2, *y*−1/2, *z*.
